# The Influence of Carbon Nanotubes on the Protective Properties of Polypyrrole Formed at Copper

**DOI:** 10.3390/ma12162587

**Published:** 2019-08-14

**Authors:** Ursula Carragher, David Branagan, Carmel B. Breslin

**Affiliations:** Department of Chemistry, Maynooth University, Maynooth, Co. Kildare 08700, Ireland

**Keywords:** corrosion protection, polypyrrole, carbon nanotubes, dodecylbenzene sulfonate, copper

## Abstract

Protective polypyrrole films doped with dodecylbenzene sulfonate (DBS) were formed at copper, while carbon nanotubes (CNT) were incorporated within the polymer films with the DBS to give PPy-DBSCNT (polypyrrole films doped with DBS and incorporated CNT). The polymer films were deposited from a 0.05 M DBS solution at a pH of 6.0 at a thin polypyrrole film doped with tartrate, which served as a stable pre-layer. Low corrosion currents of 0.12 and 0.05 μA cm^−2^ were estimated using Tafel analysis for the PPy-DBS and PPy-DBSCNT films, respectively, while a significant reduction in the concentration of Cu^2+^ ions from the corroding copper was observed for the polymer-modified copper. The corrosion protection properties were attributed to the doping of the polymer by the large and immobile DBS anions and possibly, by the larger anionic micelles that are formed at a DBS concentration of 9.8 mM in the pyrrole-containing solution. These dopants give a negatively charged surface that repels chloride anions. The additional protective properties afforded by the CNTs appear to be related to the morphology of the CNT-modified polypyrrole coatings, while the functionalized CNTs also provide a negatively charged surface.

## 1. Introduction

Conducting polymers, and in particular, polypyrrole coatings, have been used in the corrosion protection of a number of metals and alloys since DeBerry first reported the formation of polyaniline at steel [1]. Since then, polypyrrole coatings have been formed at copper, copper alloys, iron, mild steel, aluminium and aluminium alloys [2,3,4,5,6,7,8,9,10,11,12,13,14,15,16,17,18,19,20]. However, relative to inert metal substrates, such as platinum, gold or glassy carbon, the electrosynthesis is considerably more complex. This is due to the concurrent oxidation of the metal during the electropolymerization reaction, as the monomers can only be oxidised at potentials higher than about 0.60 V vs. SCE. 

Polypyrrole doped with oxalate has been used in several studies, as the oxalic acid or oxalate salt inhibits the dissolution of several metals and alloys [3,4,7,13,14]. This system has been used to deposit polypyrrole in a single step, while in some reports, an initial oxalate layer is deposited before the polymer is formed. Other salts and electrolytes, including benzoate, acetate, citrate, phosphate, salicylate and phytic solutions, have been employed [15,16,17,18,19,20,21,22,23,24,25]. Cascalheira et al. [17] have studied the formation of polypyrrole at copper from aqueous salicylate solutions using in-situ AFM measurements. Initially, a non-uniform copper–salicylate layer was deposited, which inhibits the copper dissolution reactions. Then, a thin layer of polypyrrole was deposited, which follows the irregularities in the initial copper–salicylate layer. Poly(o-toluidine) coatings have also been electrodeposited from salicylate solutions [18]. Again, it was shown that passivation of the copper surface occurs with the formation of Cu_2_O and/or a copper salicylate complex. It was found that the electropolymerization of the o-toluidine only occurred after the passivation of the copper substrate. Phthalate, saccharinate and molybdate have also been employed to deposit polypyrrole at steel substrates [22,23], while polypyrrole has been formed at iron from an ionic liquid [26]. 

In recent years, there has been considerable attention devoted to combining carbon nanotubes (CNTs) with conducting polymers, such as polypyrrole, to give composites with enhanced conducting properties and long-term stability [27,28]. These systems have been used for applications as supercapacitors [28], gas sensors [29] and as cathodes in lithium-ion batteries [30]. There are only a few publications that consider this combination as protective coatings. For example, polypyrrole/CNT coatings have been formed at carbon steel from an oxalate salt [31] and at SS304 stainless steel from sulfuric acid [32]. In both cases, it was concluded that the polypyrrole/CNT provided better corrosion protection, while Gergely [33] achieved an improvement in the protective properties of alkyd paint coatings with polypyrrole alumina composites applied to cold-rolled steel on adding CNTs.

In this study, a polypyrrole/CNT coating was deposited at pure copper and the influence of the added CNTs on the protective properties of the polymer films was evaluated. The CNTs were functionalized with anionic carboxylate groups and well dispersed in dodecylbenzene sulfonate (DBS). The DBS was selected not only to disperse the CNTs but because it is a large and immobile dopant that is not lost, with only limited exchange with the solution anions [34]. This, combined with the carboxylate groups generated at the CNTs, may facilitate the repulsion of chloride anions to give more-long-term protective polypyrrole coatings. Copper was chosen as it is widely used in industrial applications, such as heating and cooling systems, as conductors in electrical power lines, in pipelines and in electronics. In many applications, copper is exposed to salt-containing solutions and the development of protective PPy-DBS and PPy-DBSCNT coatings may be relevant in some of these applications. To the best of our knowledge, polypyrrole/CNT coatings have not been deposited at pure copper and considered as protective coatings. In addition, dodecylbenzene sulfonate is considerably different to the relatively simple oxalate, benzoate or salicylate salts that have normally been used, as it has surfactant properties and the ability to form micelles [35,36]. 

## 2. Materials and Methods

All chemicals were obtained from Sigma-Aldrich (Dublin, Ireland). The CNTs were multiwalled carbon nanotubes with a purity content >99%. The pyrrole monomer (98%) was purified by distillation prior to use. It was then stored in the dark at −20 °C between experiments. A microwave purification method was employed to purify and functionalize the CNTs. Microwave-assisted purification was carried out in a closed vessel at 180 °C in the presence of 20% hydrogen peroxide for a 30 min period. The CNTs were separated from the remaining solution in a centrifuge for 10 min and then washed thoroughly with deionised water. This was repeated a total of ten times. 

A Cu rod (99.99%, 4 mm in diameter) electrode was used for the deposition of the polymer films. The rod was encased in a Teflon holder with a copper wire threaded into the base of the sample. A flat Cu disc electrode was employed for SEM and EDX measurements. The disc and rod electrodes were polished using a 1 μm diamond polish with a Buehler micro-cloth and rinsed well with deionised water, sonicated and dried under a stream of air. A standard three-electrode electrochemical cell was used with a saturated calomel electrode (SCE) and a high-surface-area platinum wire as the counter electrode. 

A CH instruments 760c potentiostat (CH Instruments, Inc. Austin, TX, USA) was used to deposit the polypyrrole films, while voltammograms were recorded with a Solartron potentiostat Model 1287 (Solartron Analytical, Farnborough, UK) using the software package Corrware. UV–visible and fluorescence measurements were carried out using Cary 50 UV–visible and Cary Eclipse fluorescence spectrometers (Agilent, Cork, Ireland). SEM measurements were performed on a Hitachi FE–scanning electron microscope (Hitachi, Dublin, Ireland) with an Inca X–act 4.12 software package (Oxford instruments, Reading, UK). The EDX analyses were carried out using an EDX Model 51–ADD0009 (Oxford Instruments, Reading, UK). An Emitech K550X gold sputter coater (Quorum Technologies Ltd., Kent, UK) was used to deposit a thin film of gold onto the polymer samples prior to SEM analysis. A LabRAM high-resolution Raman spectrometer (HORIBA, Glasgow, Scotland) was used at 600 nm to record Raman spectra, while the FTIR data were recorded using a Perkin Elmer 2000 FTIR spectrometer (Dublin, Ireland). A Jenway 4510 conductivity meter (Farnell, Dublin, Ireland) was employed to follow the changes in the conductivity of the solution as the DBS concentration was increased. This was used to estimate the critical micelle concentration (CMC) of DBS by following the changes in the conductivity above and below the CMC. 

The DBS doped polypyrrole films were formed to give PPy-DBS, while the CNTs were incorporated to generate PPy-DBSCNT, and in both cases, these polymers were deposited onto an initial pre-layer of polypyrrole deposited from a tartrate solution. The initial PPy-Tar pre-layer was deposited from 0.1 M sodium tartrate and 0.3 M pyrrole at 0.75 V vs. SCE for 600 s. The outer PPy-DBS and PPy-DBSCNT layers were formed at 0.75 V vs. SCE with 0.3 M pyrrole and 0.05 M DBS for an additional period until a charge of 0.12 C was reached, after approximately 600–700 s. The functionalized CNTs (0.02 mg) were dispersed in 25 mL of 0.05 M DBS and then sonicated for 50 min, to give a homogeneous mixture. The pyrrole monomer was then added and the mixture was sonicated for an additional 15 min period. The pH of the DBS solution was adjusted to a pH of 6.0 and all DBS solutions were prepared freshly before each experiment. Using this approach, very good reproducibility was achieved. All experiments were repeated at least three times and the average data are presented. 

## 3. Results and Discussion

DBS is well known for its surfactant properties and its ability to disperse CNTs [27]. It was used in this study to disperse the functionalized CNTs in the pyrrole-containing electropolymerization solution. No other dopants were used, making DBS the main dopant species. As the electrolyte properties of DBS change with concentration, the DBS solution was characterised to obtain information on the critical micelle concentration (CMC) and aggregation number in the presence of pyrrole, while the CNTs were functionalized with carboxylic acid groups to aid dispersion and incorporation within the polypyrrole film.

### 3.1. Characterisation of the DBS Solution and Functionalization of CNTs

**Figure 1:**
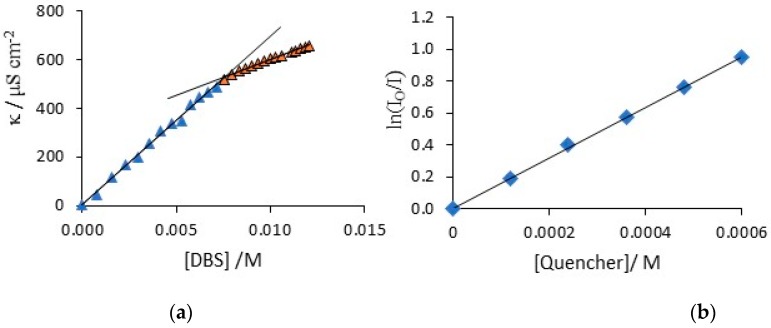
(**a**) Conductivity as a function of the dodecylbenzene sulfonate (DBS) concentration added and (**b**) fluorescence plot.

The critical micelle concentration, CMC, of DBS was determined using conductivity and fluorescence measurements. The conductivity measurements were carried out in deionised water and also in the presence of added pyrrole at 25 °C, to determine if the surfactant properties varied on adding pyrrole. In Figure 1a, a typical conductivity measurement is shown, where the conductivity is plotted as a function of the BDS concentration. Two linear regions are clearly observed. The linear region at low DBS concentrations, less than 7 mM, is associated with the DBS monomer and as more of the monomer is added, the conductivity of the solution increases in a linear manner. At these low concentrations, no micelles are formed, but at some higher concentration, the further addition of DBS gives rise to the formation of micelles, marking the CMC. As the micelles are much larger than the monomer, they have a much lower diffusion coefficient and their ionic mobility is significantly reduced to give a smaller increase in conductivity with increasing DBS concentration, which is evident at the higher DBS concentrations in Figure 1a. The intersection of these two linear segments gives an approximate critical micelle concentration of 7.8 mM. The same procedure was used to compute the CMC in the presence of 0.3 M pyrrole, which is the concentration of pyrrole used to form the polypyrrole coatings. In this case, a slightly higher CMC of 9.8 mM was computed. Fluorescence measurements are more accurate and well known [37]; however, when pyrrole is added, it is incorporated within the micelles and this complicates the fluorescence analysis. Therefore, this technique was used to validate the conductivity measurements in deionised water. The micelles were labelled with ruthenium tris bipyridine (Ru(bipy)_3_^2+^, a fluorescent probe that absorbs at a λmax of 450 nm and emits light at a λmax of 625 nm and 9-methylantracene was added as a quencher. The ratio of the luminescence intensity, I, to the solution with no quencher, I_0_, gives the CMC and N value, Equations (1) and (2). In this analysis, N is the aggregation number and it represents the number of monomers in the micelle and M represents the concentration of micelles. When the DBS concentration was maintained constant and the concentration of the quencher, Q, was varied, a linear plot was obtained. Likewise, a linear plot was obtained when the DBS concentration was varied and the quencher was maintained at a fixed concentration. A typical plot is illustrated in Figure 1b. Using these linear relationships, the average CMC was calculated as 8.1 mM and the mean N value was computed as 53. This shows that the conductivity measurements are reasonably accurate and the average CMC in the presence of pyrrole can be taken as approximately 9.8 mM with an aggregation number close to 53.

(1) IIo= exp(−[Q]/[M])

(2) $$M\;=\;\frac{\left[DBS\right]\;-\;CMC}{N}$$

**Figure 2:**
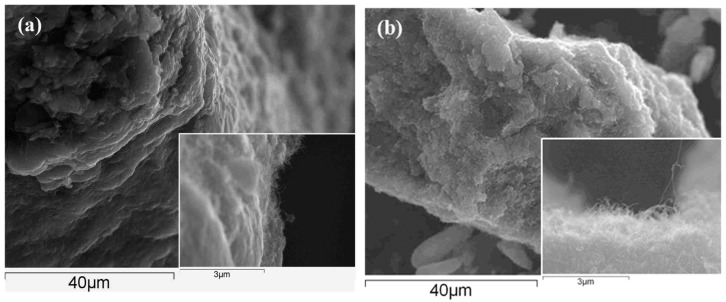
SEM micrographs of (**a**) acid functionalized and (**b**) pristine carbon nanotubes.

**Figure 3:**
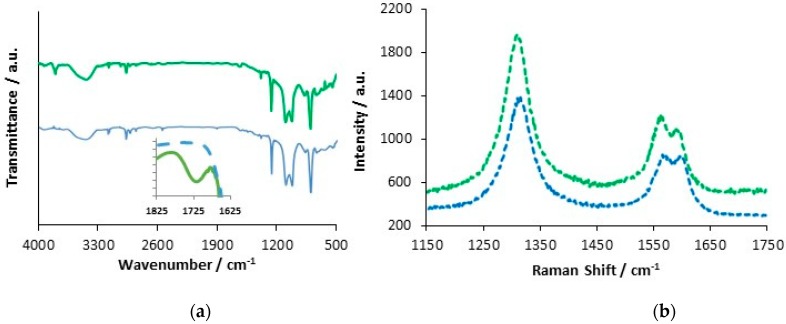
(**a**) FTIR of acid functionalized (green) and pristine carbon nanotubes (blue) (**b**) Raman spectra of acid functionalized (green) and pristine carbon nanotubes (blue).

The CNTs were functionalized as previously described and then thoroughly and repeatedly washed to remove any chemical residues. In Figure 2a,b, SEM micrographs are compared for the chemically functionalized and pristine CNTs. While the micrographs are similar in many respects, the functionalized CNTs appear to be mainly free of surface precipitates, while these are more evident for the pristine CNTs and this is consistent with the oxidation and cleaning effects. The presence of carboxylic groups on the CNTs was confirmed using FTIR, Figure 3a, where the > C=O group is seen at 1725 cm^−1^, in the inset. In both the pristine and functionalized CNTs, the C–H stretches are evident between 2960 and 2830 cm^−1^, and these are attributed to the asymmetric and symmetric CH_2_ stretching modes of the C–H bonds [38]. The broad band at about 3440 cm^−1^ can be attributed to –OH stretching, arising from the –COH and –COOH groups, while some free hydroxyls are evident at 3800 cm^−1^ for the functionalized CNTs. The peak at 1384 cm^−1^ is due to C–OH stretching vibrations and this is more pronounced with the functionalized CNTs. The resolved bands in the 1250 to 850 cm^−1^ wave region correspond to hydrated surfaces, including –OH deformation and –CO stretching, and aromatic carboxylic acids. 

Using EDX analysis, the elemental oxygen content in the sample was estimated at 1.0% for the pristine CNTs and 8.6% for the functionalized CNTs, clearly showing the incorporation of additional –COOH groups during functionalization. An acid–base titration was also carried out using standardised 0.01 M NaOH and 0.01 M HCl solutions. Two equivalence points, at pH 8.0 and 5.0, were observed for the functionalized CNTs, but only one for the pristine CNTs. The acidic group concentration was computed and using the mass of the CNTs employed in the titration, it was estimated that that the acidic group content was 6.9% by weight. The Raman spectra, presented in Figure 3b, show the typical D and G bands and a D’ band [39]. These bands are slightly red shifted in the case of the functionalized CNTs, with the G band at 1575 cm^−1^ for the pristine CNTs and at 1568 cm^−1^ for the functionalized CNTs. The D:G ratio for the pristine CNTs was 1.9:1.0, indicating some surface manufacturing damage. This ratio increased to 2.5:1.0 for the functionalized CNTs, consistent with the introduction of the acidic groups.

### 3.2. Formation PPy-DBS and PPy-DBSCNT

**Figure 4:**
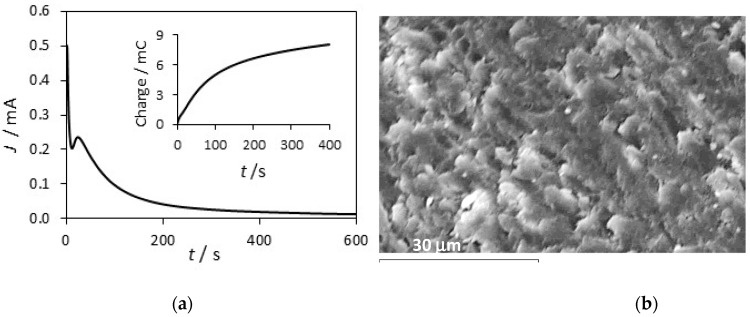
(**a**) Current-time and charge (inset) plots for the formation of PPy-Tar at copper in 0.3 M pyrrole and 0.1 M sodium tartrate at 0.75 V vs. SCE, (**b**) SEM micrographs recorded for PPy-Tar.

The PPy-DBS and PPy-DBSCNT polymer films were deposited at an initial PPy-Tar pre-layer that forms readily at the copper substrate [34]. This initial layer was deposited as the direct formation of polypyrrole from DBS is difficult to achieve. Dissolution of copper occurs and the formation of a passive layer is slow to form in the DBS solution [34]. The electropolymerization of pyrrole in tartrate is illustrated in Figure 4a, where the current–time plot is shown for the formation of polypyrrole at copper at 0.75 V vs. SCE in 0.3 M pyrrole with 0.10 M sodium tartrate. The charge–time curve shown in the inset deviates significantly from the normal near-linear plot expected for efficient electropolymerization. The higher charges seen during the first 100 s are consistent with the initial dissolution of copper, while the lower rate of charge increase after the elapse of this initial dissolution phase is associated with passivation of the copper in the tartrate solution and the formation of the polymer. The corresponding SEM micrograph is presented in Figure 4b. Polypyrrole nodules are apparent; however, the typical cauliflower structure is not evident and this is most likely due to the thin layer of polypyrrole deposited and the underlying copper–tartrate. The thickness of the initial PPy-Tar layer was estimated as 0.7 ± 0.3 µm from the cross-section SEM observation of the film.

**Figure 5:**
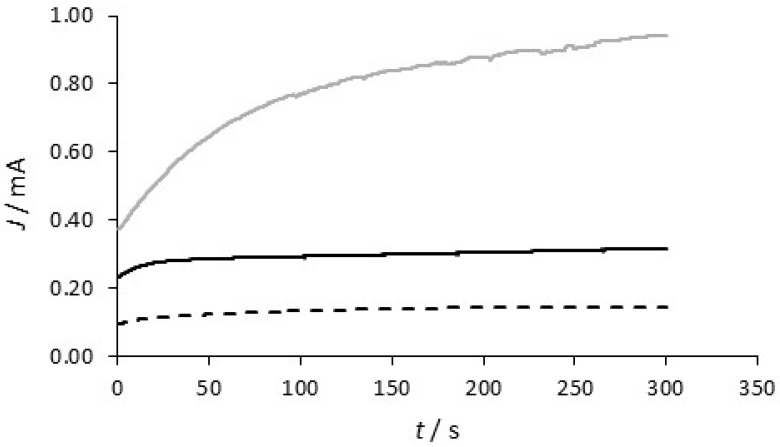
Current–time plots for the formation of PPy-DBS at **- - -** 0.75 V and ▬▬▬ 0.90 V vs. SCE and ▬▬ PPy-DBSCNT at 0.75 V at a PPy-Tar layer on copper.

In Figure 5, the electropolymerization of pyrrole in DBS at this initial PPy-Tar layer is shown in the presence and absence of the carbon nanotubes. Initially, the concentration of DBS was varied and it was found that a 0.05 M concentration enabled efficient electropolymerization and provided the more protective polymer films [34]. For this second polymer layer, the current is initially low and then increases over the time period. The current increases at a faster rate when the DBS-containing polymer is formed at 0.90 V vs. SCE. This is consistent with an increasing rate of electropolymerization at the higher applied potential and a somewhat higher surface area as the polymer is formed on the stable PPy-Tar layer. A lower rate of electropolymerization is evident in the presence of DBS at the lower potential of 0.75 V vs. SCE; however, it is clear from this plot that the near constant current indicates very good stability of the underlying PPy-Tar layer. The influence of the CNTs is evident on comparing the rates of electropolymerization at 0.75 V vs. SCE. There is a two-fold increase in the current in the presence of the carbon nanotubes. The functionalized carbon nanotubes may act as dopants, but they are also incorporated within the polymer matrix, providing conducting scaffolds for further polymer growth. These data are consistent with this additional conducting interface that facilities the growth of the polymer, to give higher rates of electropolymerization. 

**Figure 6:**
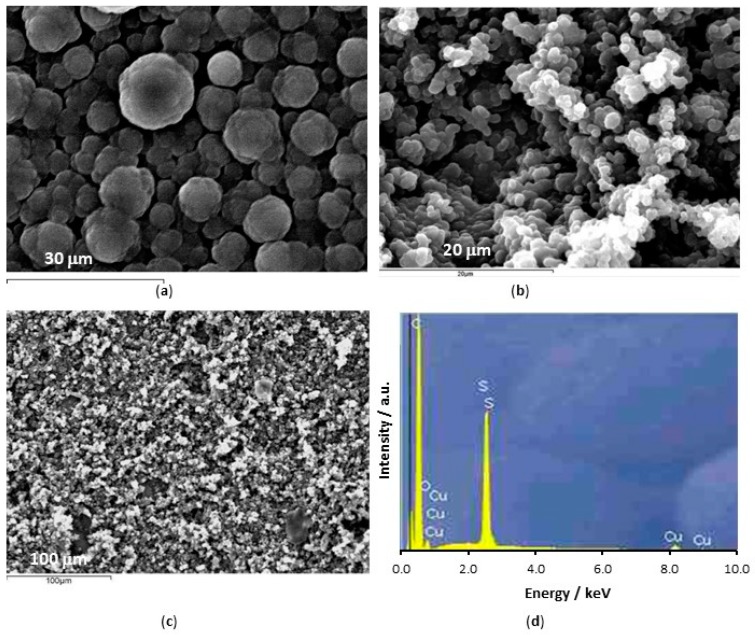
SEM micrographs recorded for (**a**) PPy-DBS, (**b**) PPy-DBSCNT, (**c**) PPy-DBSCNT at lower magnification and (**d**) EDX spectrum recorded for PPy-DBSCNT.

The surface morphologies of the PPy-DBS and PPy-DBSCNT films deposited at PPy-Tar are shown in Figure 6 and it is clear that the CNTs have an influence on the nature and morphology of the surface. The typical cauliflower-like structures are clearly evident in the absence of the CNTs, Figure 6a, and the size of the cauliflower-like structures reach diameters of about 15 µm. This surface morphology is very different to that seen with the PPy-Tar, Figure 4b, indicating efficient nucleation of the PPy-DBS at the PPy-Tar pre-layer. The cauliflower-like structures appear considerably smaller in the presence of the CNTs and the polymer adopts a more disorganised structure, see Figure 6b. This is consistent with the incorporated CNTs providing a scaffold or backbone for the further growth of the polymer, with the polymer forming bridges between the CNTs. As shown in Figure 6c, the carbon nanotubes are well dispersed throughout the polymer, with evidence of only small amounts of agglomeration. While the CNTs may act as dopants due to the carboxylic acid groups, these anionic −COO^−^ groups will be in equilibrium with −COOH and as the interfacial pH becomes more acidic as the electropolymerization reaction occurs, the equilibrium will shift towards the −COOH, making the CNTs less likely to dope the polymer. Therefore, it is probable that some or indeed many of the incorporated CNTs are not dopants and when the polymer is immersed in a neutral solution, the neutral –COOH groups will be ionised to give anionic −COO^−^ groups that are free and not involved in charge compensation. This that may help to repel the chloride anions from the polymer–solution interface. The EDX spectrum presented in Figure 6d clearly shows the presence of S, indicating the presence of DBS as a dopant. Nevertheless, it is possible that some micelles are incorporated within the polymer matrix. The DBS concentration of 0.05 M is well above the CMC value of approximately 9.8 mM and this means that the DBS will exist not only as monomers but also as micelles in solution, with the hydrophobic chain located in the core of the micelle, and the negatively charged SO_3_^−^ groups pointing outwards into the solution. These negatively charged micelles could also be incorporated as dopants during the formation of the polymer.

### 3.3. Corrosion Protection Properties

**Figure 7:**
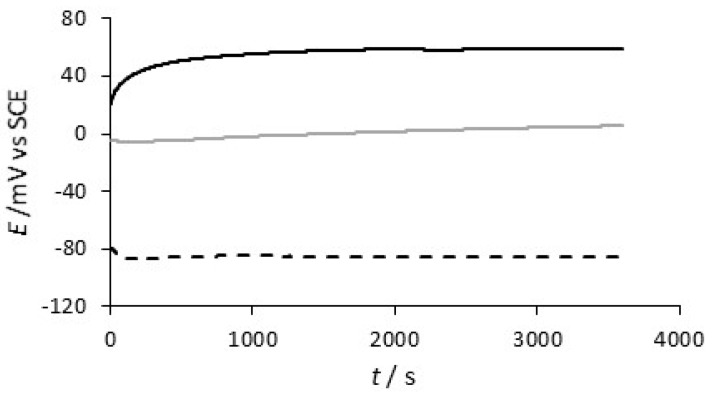
Open-circuit potential plotted as a function of time for **- - -** Cu, ▬ PPy-DBS and, ▬ PPy-DBSCNT recorded in a neutral 0.1 M NaCl.

The corrosion protection properties were studied using a combination of slow scan rate voltammograms, analysis of the copper concentrations released following days of immersion in 0.1 M NaCl, open–circuit potential measurements and Tafel analysis. As the rate of electropolymerization is higher with the CNTs, Figure 5, the final PPy-DBS and PPy-DBSCNT layers were deposited to the same final charge of 0.12 C. The open–circuit potentials of uncoated copper, PPy-DBS and PPy-DBSCNT are shown in Figure 7. These data were recorded over a 20-day period and the data shown in Figure 7 correspond to the first 600 min. The open-circuit potential adopted by the copper electrode decays to about −0.20 V vs. SCE after 12 h and then fluctuates between −0.10 V and −0.20 V vs. SCE. The open-circuit potentials of the two polymer systems remain essentially constant and higher than the potential of the copper electrode over the 20-day period. The open-circuit potential of the PPy-DBS system varied between −0.05 V and 0.15 V vs. SCE, while the PPy-DBSCNT coatings maintained a slightly higher potential of 0.05 V to 0.25 V vs. SCE over the 20-day period, indicating good protective properties. 

**Figure 8:**
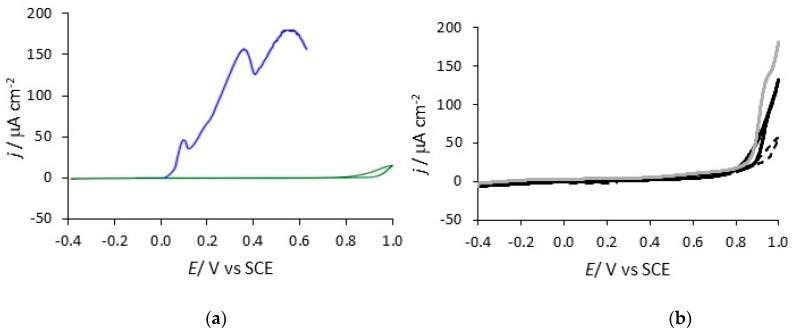
Voltammograms recorded at 1 mV s^−1^ in 0.1 M NaCl, at pH 7.0 for (**a**) ▬▬ copper and ▬▬ PPy-DBSCNT (first cycle) and (**b**) ▬ ▬ PPy-DBS (first cycle), ▬▬ PPy-DBS (second cycle) and ▬▬ PPy-DBSCNT (second cycle).

Cyclic voltammograms recorded at 1.0 mV s^−1^ for PPy-DBS and pure uncoated copper are shown in Figure 8. The onset of copper dissolution in this chloride-containing solution is seen at about 0.0 V vs. SCE and the current then continues to increase and fluctuate as the copper is polarised to higher potentials and corrosion products are deposited at the surface. The PPy-DBSCNT-coated electrode was cycled from −0.40 V to an upper potential limit of 1.0 V vs. SCE, and there is very little evidence for the dissolution of the copper substrate, which indicates the good protective properties of the PPy-DBSCNT coatings. Similar protective properties were seen with PPy-DBS and the corresponding slow scan voltammograms are illustrated in Figure 8b. While the currents are somewhat lower between 0.85 and 1.0 V vs. SCE for the PPy-DBSCNT system, these potentials will not be reached in any real application and the currents are nearly identical at lower potentials in the vicinity of 0.0 to 0.40 V vs. SCE. When the PPy-DBSCNT-coated copper was cycled back to −0.40 V vs. SCE and then cycled in the forward direction, for a second time, breakdown of the coating was observed at about 0.80 V vs. SCE, indicating some loss in the protective properties. A similar trend is evident with PPy-DBS, as shown in the voltammograms for the second cycles, see Figure 8b. This indicates that the dissolution observed during the first cycle is not repaired once the copper substrate begins to dissolve, and there is some loss in the protective properties of the polymer. It is clear from these data that the polymer provides good protection to the underlying copper substrate. The current remains low, typically at 1 to 2 μA until the potential reaches about 0.80 V vs. SCE and then dissolution of copper becomes evident. 

**Figure 9:**
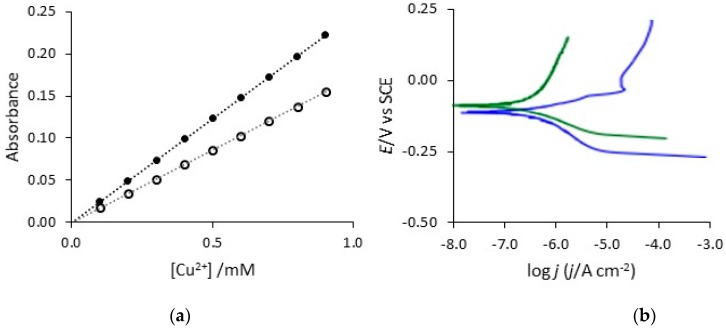
(**a**) Calibration curve recorded in the ○ absence and ● presence of 0.1 M NaCl at a pH of 7.0 and (**b**) Tafel plots recorded for ▬▬ PPy-DBSCNT and ▬▬ scratched PPy-DBSCNT.

As detailed earlier, the potentials reached in Figure 8 are not representative of any real application and, therefore, the corrosion protection properties of the polymer coatings were assessed by following the concentration of Cu^2+^ released from the electrodes immersed under open-circuit potential conditions in 0.1 M NaCl. In order to determine the concentration of copper dissolved from the polymer-modified copper electrodes, a spectrophotometric method involving the bathocuproine chelating agent [40] was used. This analytical approach is normally used in the absence of chloride, but as shown in the calibration curves depicted in Figure 9a, a linear calibration curve was generated in the presence of 0.1 M NaCl on recording the absorbance at 480 nm. Although a higher gradient was observed in the presence of chloride, which is consistent with the participation of the chloride anions in the reaction, as shown in Equation (3), the absorbance can be used to give the concentrations of Cu^2+^ generated, provided the chloride concentration is fixed.

Cu^2+^ + nCl^−^ → CuCl_n_^n−1^ + (bathocuproine) → Cu(bathocuproine)Cl + (n − 1) Cl^−^ (3)

In Table 1, the concentrations of Cu^2+^ recorded following two days of immersion in 0.1 M NaCl are summarised. For comparative properties, the protective properties of the underlying PPy-Tar system were also monitored. Furthermore, as the surface morphology of the PPy-DBS and PPy–DBSCNT are different, as illustrated in Figure 6, the fixed charge of 0.12 C may not result in the same polymer thickness and, therefore, the PPy-DBS was deposited to charges of 0.12 C and 0.18 C to determine if a small change in the polymer thickness or charge influenced the protective properties. It is evident from these data that the PPy-DBSCNT and PPy-DBS provide the more protective polymer coatings, with the lowest concentrations of dissolved copper. There is very little difference between the PPy-DBS films formed at 0.12 C and 0.18 C. It is also evident that the initial layer of PPy-Tar has a much lower protective property, when exposed to a chloride-containing solution. However, the concentrations provided in Table 1 may be influenced by the deposition of copper-containing corrosion products, particularly at the uncoated copper electrode. In the presence of chloride anions, CuCl and other CuCl_n_^n−1^ species, are deposited at the surface, and while these precipitates may have very poor corrosion protection properties, the porous nature of the film may limit the true concentration of copper released into the solution, giving solution concentrations that are lower than the true rate of dissolution. These corrosion products were not observed with the polymer-coated copper electrodes and the concentrations of dissolved copper are a more realistic measure of the true corrosion rate and the protective properties of the polymer systems. 

Tafel analysis was carried out to estimate the corrosion current. As the displacement from the corrosion potential, E_corr_, is increased, a logarithmic relationship between the potential and the current is observed, to give the Tafel equation, Equation (4).

(4) $$E-E_{\mathrm{corr}}\;=\;\frac{2.303\mathrm{RT}}{\alpha F}{\mathrm{logj}}_{\mathrm{corr}}-\frac{2.303\mathrm{RT}}{\alpha F}\mathrm{logj}$$

In this analysis, j_corr_ is the corrosion current density, j is the measured current density, α is the transfer coefficient and the term E − E_corr_ represents the overpotential. It is possible to extrapolate the anodic and cathodic linear portions of the polarization curves to give E_corr_ and the magnitude of the current at this point gives j_corr_. A typical polarization curve for PPy-DBSCNT, recorded at a scan rate of 0.1667 mV s^−1^ in an attempt to reduce the charging current contribution from the conducting polypyrrole, is shown in Figure 9b. The computed E_corr_, j_corr_ and cathodic Tafel slope values are summarised in Table 2 for pure copper, PPy-Tar, PPy-DBS and PPy-DBSCNT. The computed j_corr_ value obtained for copper is in relatively good agreement with the data obtained by Mansfeld et al. [41] for copper in chloride solutions. It is clear that higher j_corr_ values are computed for the uncoated copper and PPy-Tar-modified copper and considerably lower values are obtained for the polymers containing DBS and CNTs, which is consistent with the data presented in Table 1. Interestingly, the cathodic Tafel slopes are somewhat higher for the PPy-DBSCNT polymer system with no evidence to show that the conducting CNTs facilitate the reduction reaction. In a corrosion reaction at a near-neutral pH, this reduction is normally the reduction of dissolved oxygen. This may be an advantage as any enhancement in this reduction reaction could facilitate corrosion of the copper substrate. It appears that the CNTs are either wrapped within the forming polymer and the conducting CNT surface is blocked and not available to support the reduction of dissolved oxygen, or the CNTs are too far removed from the copper substrate to provide the reduction half reaction.

The protective properties and stability of PPy-DBS and PPy-DBSCNT were studied further using a scratch test. A small scratch was made in the coatings and then the polarization behaviour of the scratched coating was studied. A typical plot is presented in Figure 9b, where the scratched PPy-DBSCNT is compared to the protective coating. It is evident on a comparison of the two plots in Figure 9b that the scratched polymer has a higher j_corr_ value and a clear anodic peak is observed at about 0.0 V vs. SCE, indicating dissolution of the copper substrate. There is also a shift in E_corr_ to lower values, which are more typical of the uncoated copper electrode. Similar results were obtained with PPy-DBS. This analysis shows that while the intact polymer coatings have good protective properties, once the coating is damaged and the underlying copper substrate is exposed to the aggressive chloride solution, the polymer is no longer able to maintain good protective qualities. 

### 3.4. Role of CNT, DBS and Tartrate Pre-Layer

Although the PPy-DBS and PPy-DBSCNT polymer films behave similarly, somewhat lower corrosion currents and lower amounts of released Cu^2+^ are observed with PPy-DBSCNT. The conducting CNTs provide a conducting scaffold for the formation and nucleation of polypyrrole and this modifies the structure of the polymer, as evident in Figure 6, and gives rise to higher rates of electropolymerization, see Figure 5. While it is difficult to ensure that both the PPy-DBS and PPy-DBSCNT polymer films have similar thickness, increasing the electropymerization time to give a final charge of 0.18 C compared to 0.12 C for the PPy-DBS system had little influence on the protective properties, as illustrated in Table 1 and Table 2. This suggests that the CNTs enhance somewhat the protective properties of the DBS-doped polymer. In terms of doping, the polymer may be doped with both individual DBS anions, the larger anionic micelles, and to a lesser extent, with the CNTs to give a negatively charged surface that repels chloride anions. Furthermore, the negatively charged −COO^−^ groups on the CNTs that are free and not involved in charge compensation within the polymer matrix will also repel chloride anions. Support for the additional incorporation of micelles comes from observations made by Bay et al. [42], who observed a large increase in the doping levels when alkyl benzenesulfonates, such as DBS, were doped within polypyrrole, while Prissanaroon et al. [43] observed doping levels of 0.55 for DBS-doped polypyrrole films.

The potentials adopted by the PPy-DBS-coated and PPy-DBSCNT-coated copper are close to the potential for the formation of Cu_2_O, Equation (5), and this indicates that the copper substrate can be maintained as the Cu(I) oxide/hydroxide. However, in the presence of chloride anions, CuCl and other CuCl_n_^n−1^ species are generated, giving rise to corrosion.

Cu_2_O + 2H^+^ + 2e^−^ → 2Cu + H_2_O  E = 0.471−0.0591 pH (5)

Therefore, the anionic DBS, micelles and functionalized CNTs that are large and immobile are important elements as they provide a negatively charged surface that repels the chloride anions. While equilibrium exchange between small mobile dopants and chloride anions takes place at the polymer solution interface, the DBS and CNT dopants will remain incorporated in the outer polymer layer. It is well known that reduction of the polymer occurs on dissolution of the copper substrate. While the dopants in the outer layer are immobile, the reduction of the initial PPy-Tar pre-layer, will result in the release of the more mobile tartrate anions, which may lead to repassivation of the corroding substrate. This inner PPy-Tar layer also serves to reduce the mobility of Na^+^ cations that are incorporated at the outer layer, when the polymer is reduced. It has been shown by Rohwerder and Michalik [44] that cation transport is fast in polypyrrole films doped with immobile anions, while structural damage within the polypyrrole matrix [45] has been observed on the ingress of Na^+^ and its associated solvated water molecules. As the inner PPy-Tar is reduced to the neutral polymer with the release of the tartrate anions, the transport of cations within this inner layer is considerably reduced, providing better polymer stability and minimising the risk of polymer delamination at the corroding site. This inner PPy-Tar layer also provides a barrier between the CNTs and the copper substrate, preventing any local corrosion cell that might arise between the corroding copper substrate and the reduction of oxygen at the conducting CNTs. 

## 4. Conclusions

Polypyrrole films doped with dodecylbenzene sulfonate and with incorporated CNTs were deposited at an initial polypyrrole film doped with a tartrate pre-layer to give protective coatings for the corrosion protection of copper. This was evident from open-circuit potential measurements where the open-circuit potential remained constant and higher than the uncoated copper for periods in excess of 20 days. Breakdown potentials of approximately 0.85 V vs. SCE were obtained on cycling the polymer-coated electrodes in 0.1 M NaCl, while the corrosion currents were reduced from 1.95 μA cm^−2^ to 0.12 μA cm^−2^ for PPy-DBS and to 0.05 μA cm^−2^ for PPy-DBSCNT. The corrosion protective properties were explained in terms of the large and immobile DBS, functionalized CNTs and possibly additional anionic micelles that repel the chloride anions. While the functionalized CNTs provide a negatively charged surface, they also give a highly conducting phase that enables higher electropolymerization rates and the deposition of thicker polymer films at shorter time periods. 

## Figures and Tables

**Table 1:** Concentration of Cu^2+^ measured following a 2-day immersion period in 0.1 M NaCl.

System	Concentration/mM
Uncoated Cu	4.95 ± 0.15
PPy-Tar PPy-DBS (0.12 C) PPy-DBS (0.18 C)	1.93 ± 0.17 0.62 ± 0.10 0.59 ± 0.10
PPy-DBSCNT	0.50 ± 0.10

**Table 2:** Tafel slopes, *E*_corr_ and *I_c_*_orr_ estimated from polarization curves.

System	*b*_c_/mV Decade^−1^	*E*_corr_ V vs. SCE	*I*_corr_ µA cm^−2^
Uncoated Cu	95	−0.235 ± 0.015	1.95 ± 0.03
PPy-Tar	52	−0.080 ± 0.030	1.70 ± 0.04
PPy-DBS (0.12 C)	53	−0.065 ± 0.017	0.12 ± 0.06
PPy-DBS (0.18 C)	55	−0.060 ± 0.018	0.12 ± 0.05
PPy-DBSCNT	70	−0.070 ± 0.016	0.05 ± 0.06
